# Hexobarbital Sleep Test for Predicting the Susceptibility or Resistance to Experimental Posttraumatic Stress Disorder

**DOI:** 10.3390/ijms21165900

**Published:** 2020-08-17

**Authors:** Maria Komelkova, Eugenia Manukhina, H. Fred Downey, Alexey Sarapultsev, Olga Cherkasova, Viacheslav Kotomtsev, Pavel Platkovskiy, Stanislav Fedorov, Petr Sarapultsev, Olga Tseilikman, David Tseilikman, Vadim Tseilikman

**Affiliations:** 1 School of Medical Biology, South Ural State University, 454080 Chelyabinsk, Russia; mkomelkova@mail.ru (M.K.); manukh@mail.ru (E.M.); Fred.Downey@unthsc.edu (H.F.D.); diol2008@yandex.ru (O.T.); vadimed@yandex.ru (V.T.); 2 Institute of Immunology and Physiology, Ural Division of the Russian Academy of Sciences, 620049 Ekaterinburg, Russia; 2134012@gmail.com (V.K.); p.sarapultsev@gmail.com (P.S.); 3 Laboratory for Regulatory Mechanisms of Stress and Adaptation, Institute of General Pathology and Pathophysiology, 125315 Moscow, Russia; 4 Department of Physiology and Anatomy, University of North Texas Health Science Center, Fort Worth, TX 76107, USA; 5 Biophysics Laboratory, Institute of Laser Physics, Siberian Branch of the Russian Academy of Science, 630090 Novosibirsk, Russia; cherk_63@mail.ru; 6 Laboratory of Biomedical Research, Ural Research Institute for Phthisiopulmonology of Ministry of Health of Russian Federation, 620039 Ekaterinburg, Russia; 7 Department of Fundamental Medicine, Chelyabinsk State University, 454001 Chelyabinsk, Russia; f.schwarcz@yandex.ru (P.P.); fedorovstas2016@yandex.ru (S.F.); tceilikmanve@susu.ru (D.T.)

**Keywords:** hexobarbital sleep test, posttraumatic stress disorder, corticosterone, glucocorticoid metabolism, resilience

## Abstract

Hexobarbital sleep test (HST) was performed in male Wistar rats (hexobarbital 60 mg/kg, i.p.) 30 days prior to stress exposure. Based on the duration of hexobarbital-induced sleep, rats were divided into two groups, animals with high intensity (fast metabolizers (FM), sleep duration <15 min) or low intensity of hexobarbital metabolism (slow metabolizers (SM), sleep duration ≥15 min). The SM and FM groups were then divided into two subgroups: unstressed and stressed groups. The stressed subgroups were exposed to predator scent stress for 10 days followed by 15 days of rest. SM and FM rats from the unstressed group exhibited different behavioral and endocrinological patterns. SM showed greater anxiety and higher corticosterone levels. In stressed animals, anxiety-like posttraumatic stress disorder (PTSD) behavior was aggravated only in SM. Corticosterone levels in the stressed FM, PTSD-resistant rats, were lower than in unstressed SM. Thus, HST was able to predict the susceptibility or resistance to experimental PTSD, which was consistent with the changes in glucocorticoid metabolism.

## 1. Introduction

Posttraumatic stress disorder (PTSD) is a severe mental disorder that develops in people exposed to trauma [1]. PTSD is a delayed response to a traumatic event following a prolonged latent period [1,2]. This latent period provides an opportunity for treatment to lessen subsequent PTSD or perhaps even prevent it. For this approach to be efficient, it would be valuable to predict which persons exposed to trauma are PTSD-susceptible, but at present, this capability does not exist. Importantly, only about 20% of traumatized subjects develop PTSD [3]. Therefore, people may be PTSD-susceptible or PTSD-resistant.

Recently, we showed that a hexobarbital sleep test (HST) could predict in which rats a halogenated glucocorticoid, triamcinolone acetonide, would produce a side effect of elevated anxiety [4]. From the duration of hexobarbital-induced sleep, the HST identified rats with high or low intensity of microsomal oxidation (fast or slow metabolizers, FM, or SM) and, thus, predicted their anxiety response to the exogenous glucocorticoid. Another known side effect of this drug is the suppressed production of endogenous glucocorticoids due to adrenal gland dystrophy [5]. The development of PTSD symptoms, including anxiety [1], is preceded by prolonged, stress-induced exposure to high levels of glucocorticoids [6], which eventually results in exhaustion and dystrophy of the adrenal glands and decreased glucocorticoid production [7].

Since the consequences of exogenous and stress-induced high concentrations of glucocorticoids are similar, it appeared that the HST might be capable of predicting individual rat vulnerability or resistance to PTSD. Our approach was to compare the behavioral and hormonal responses of rats exposed to traumatic stress with their hexobarbital sleep time.

Earlier, we showed that FM and SM phenotypes differ in the intensity of microsomal oxidation and the resulting levels of glucocorticoids [4,8]. Glucocorticoids are metabolized via two major pathways, by cytochrome P4503A (CYP3A), which depends on microsomal oxidation) and by 11β-hydroxysteroid dehydrogenase type 2 (11βHSD-2), which does not depend on microsomal oxidation). However, to demonstrate that these phenotypes were, indeed, present in this study and associated with vulnerability or resistance to PTSD, we confirmed that FM and SM rats used different metabolic pathways to metabolize glucocorticoids. For this reason, not only corticosterone, but also the markers of CYP3A-dependent and 11βHSD-2 dependent glucocorticoid metabolic pathways, were measured.

## 2. Results

### 2.1. Effect of Microsomal Oxidation Phenotype and Predator Stress on Rats Behavior

Behavioral data are reported in Table 1. Two-way ANOVA showed the effect of the microsomal oxidation phenotype on the behavior of FM and SM rats. The presence of statistically significant differences in the time spent in the open (F1.62 = 22.86: *p* < 0.001) and closed (F1.62 = 22.86: *p* < 0.001) arms, as well as on the number of entries into the open (F1.62 = 19.88: *p* < 0.001) and closed (F1.62 = 3.95: *p* < 0.05) arms of the elevated plus-maze, was revealed. In unstressed FM rats, the time spent and the number of entries into open arms were higher than in unstressed SM rats (*p* = 0.038 and *p* = 0.025, respectively). On the contrary, the time spent and the number of entries into closed arms in unstressed FM were lower in comparison to unstressed SM rats (respectively, *p* = 0.038 and *p* = 0.034).

According to two-way ANOVA, stress had a significant impact on the time spent in the open (F1.62 = 6.63: *p* = 0.012) and in closed (F1.62 = 6.95: *p* = 0.011) arms, as well as on the number of entries into the open (F1.62 = 6.63: *p* = 0.012) and closed (F1.62 = 22.34: *p* < 0.001) arms of the elevated plus-maze. Stressed FM rats have revealed an increase in time spent (*p* < 0.001) and a number of visits (*p* = 0.002) into open arms, and the decrease in time spent in closed arms (*p* < 0.001) of the elevated plus-maze in comparison to control FM animals. Stressed FM and SM rats have displayed an increase in the number of entries into closed arms compared with the corresponding controls (*p* = 0.003 and *p* < 0.001) was also detected in stressed FM and SM rats.

The significant effect of the microsomal oxidation phenotype on the anxiety index (AI) value (F1.62 = 22.33: *p* < 0.001) was revealed. The unstressed FM rats had lower AIs in comparison to unstressed SM rats (*p* = 0.04) and stress further reduced AIs in FM rats (*p* = 0.003) with no effect on SM rat’s AIs. The AI significantly correlated with HST time (r = 0.83; *p* < 0.01; *n* = 62).

According to two-way ANOVA, there were no significant differences in grooming (F1.62 = 3.45: *p* = 0.07), but the clear upward trend toward an increase in the number of grooming acts was detected in stressed SM rats.

### 2.2. Effect of Microsomal Oxidation Phenotype and Predator Stress on Plasma Corticosterone Concentration

According to two-way ANOVA, the FM or SM phenotype exerted significant effects on concentrations of blood corticosterone (F1.62 = 4.38: *p* = 0.041, Table 1). Concentrations of corticosterone were higher in unstressed SM than unstressed FM rats (*p* = 0.028).

Two-way ANOVA showed that stress exerted significant effects on corticosterone (F1.62 = 16.37: *p* < 0.001). Plasma concentration of corticosterone was lower both in stressed FM (*p* = 0.019) and SM (*p* = 0.002) rats in comparison to control animals. 

Moreover, plasma corticosterone concentration significantly correlated with the number of entries in open arms (r = ‒0.65 *p* < 0.05; *n* = 8) and with grooming (r = 0.79; *p* < 0.05; *n* = 8) in unstressed SM rats.

### 2.3. Effect of Microsomal Oxidation Phenotype and Predator Stress on Corticosterone Metabolites Concentrations

According to two-way ANOVA, microsomal oxidation phenotype exerted significant effects on corticosterone metabolites concentrations (i.e., 11DehydroCort in blood (F1.62 = 10.98: *p* = 0.002) and 6βCort in liver (F1.62 = 7.19: *p* = 0.01) (Table 1). 

Concentration of 11Dehydro Cort was higher in unstressed SM than unstressed FM rats (*p* = 0.009). The hepatic level of 6βCort was lower in unstressed SM than unstressed FM rats (*p* = 0.002). Plasma 11Dehydro Cort concentration significantly correlated with number of entries in close arms (r = ‒0.94 *p* < 0.05; *n* = 8), with grooming (r = 0.91; *p* < 0.05; *n* = 8) in unstressed SM rats. Hepatic 6βCort significantly correlated with grooming (r = ‒0.45 *p* < 0.05; *n* = 22) in unstressed FM rats.

Two-way ANOVA showed, that stress exerted significant effects on 6βCort (F1.62 = 7.69: *p* = 0.009) concentration in blood. In FM animals, stress caused an increase in 6βCort concentration in blood (*p* = 0.004). With that, stress did not affect the concentration of 11Dehydro Cort in the blood of rats with SM and FM phenotypes. 

Thus, it can be concluded that CYP3A-dependent glucocorticoid metabolism predominated in FM rats, while 11βHSD-dependent metabolism predominated in SM rats. Exposure to stress did not cause any fundamental changes in the type of glucocorticoid metabolism.

### 2.4. Effect of Microsomal Oxidation Phenotype and Predator Stress on Kidneys 11βHSD-2 Activity

According to two-way ANOVA, both phenotypes exerted significant effects on kidney 11βHSD-2 (F1.62 = 7.23: *p* = 0.009). Kidney 11βHSD-2 activity was higher in unstressed SM than unstressed FM rats (*p* = 0.01). Notably that kidney 11βHSD-2 activity significantly correlated with times spent in open arms (r = ‒0.94 *p* < 0.01; *n* = 8), with times spent in closed arms (r = 0.91; *p* < 0.01; *n* = 8) with number of entries in the open arms (r = ‒0.83 *p* < 0.05; *n* = 8) and with AI (r = 0.94; *p* < 0.01; *n* = 8) in unstressed SM rats.

## 3. Discussion

Previously, predator scent stress (PSS) has been shown to produce PTSD anxiety-like behavior in rats [9]. In the current study, rats were divided into two groups, animals with high intensity (fast metabolizers, FM, sleep duration <15 min) or with low intensity of hexobarbital metabolism (slow metabolizers, SM, sleep duration ≥15 min), and, according to the results, the unstressed SM rats had an average AI = 0.71, whereas the AI of unstressed FM had an average AI = 0.65. Moreover, stress has further reduced AIs in FM rats (*p* = 0.003) with no effect on SM rat’s AIs. Thus, one can conclude that HST was able to reveal rats predisposed to PTSD.

PPS has led to a decrease in corticosterone levels in both phenotypes. This is consistent with previously reported results, where a similar decrease in the blood corticosterone in rats exposed to traumatic stress was observed [7].

Importantly, unstressed SM and FM rats differed in their glucocorticoid metabolism. Unstressed FM had a lower level of corticosterone than SM rats, which was consistent with our previous data [4,8]. Effects of PTSD on plasma corticosterone/cortisol have been studied on different PTSD models and in clinical studies. Some PTSD models induced an increase in plasma corticosterone, such as single and done-twice predator stress [10,11]. Furthermore, Wilson et al. (2013) observed both elevated adrenal corticosterone production and adrenal gland hypertrophy [12]. Other studies have reported reduced plasma concentrations of corticosterone in rats and of cortisol in humans, which is considered an important marker of PTSD [13,14]. In our study, experimental PTSD was induced by chronic predator stress with multiple stress exposures rather than single acute exposure, which was apparently the reason for reduced plasma corticosterone.

In the present study, we addressed the influence of the CYP3A-dependent part of glucocorticoid metabolism activity on the formation of susceptibility or resistance to experimental PTSD. Previously, it was shown that fast metabolizers (FM) are characterized by an increased level of CYP3A activity [4]. However, the method used for determining the enzymatic activity did not involve a direct evaluation of glucocorticoid metabolites, which reduced its information value. The direct evaluation of 6βCort allowed us to evaluate the glucocorticoid metabolism by the CYP3A pathway. With that, the more pronounced decrease in corticosterone in stressed SM rats was observed in comparison to FM rats. This pronounced decrease was due to the initially higher basal levels of the 11βHSD-2 and moderate activation of the CYP3A-dependent pathway enzymes, which was confirmed by the elevated levels of 6βCort in the liver.

**Figure 1:**
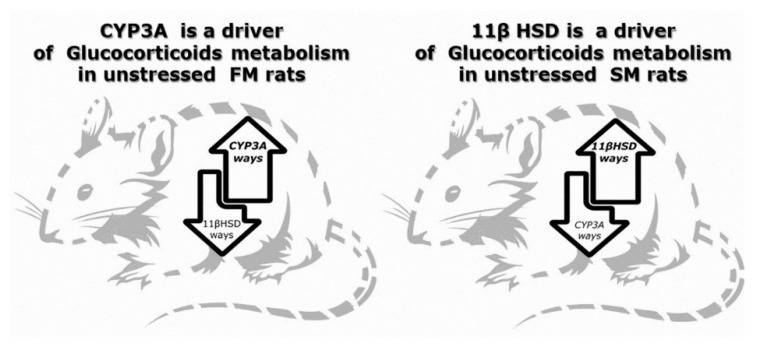
Key pathways of glucocorticoid tissue metabolism of in fast (FM) and slow (SM) metabolizers.

At the same time, the unstressed SM rats had higher levels of 11Dehydro Cort, which indicated the activation of 11βHSD-dependent glucocorticoid metabolism, as 11Dehydro Cort is a product of glucocorticoid C11-oxidation (Figure 1).

The unstressed FM and SM rats differed in the intensity of CYP3A and 11βHSD-2 reactions, since CYP3A is an enzyme of the irreversible, whereas 11βHSD-2 is an enzyme of the reversible pathway of glucocorticoid inactivation, and can be involved in the regulation of glucocorticoid recovery via 11βHSD-1 [15,16]. With that, PTSD is known to be characterized by an activated 11βHSD-2 pathway of corticosterone metabolism [2], and thus this feature of glucocorticoid metabolism, along with more pronounced anxiety points at “slow metabolism”, as a risk factor for PTSD.

In general, the initially low glucocorticoid level in FM rats may contribute to the development of resistance to behavioral disorders [17,18]. It is noteworthy that SM rats with an initially higher level of corticosterone demonstrate higher anxiety and, accordingly, are more prone to the development of behavioral disorders. With that, glucocorticoids are inducers of CYP3A expression and therefore, it is paradoxical that despite the higher basal level of corticosterone, unstressed SM rats are not characterized by the activation of the CYP3A pathway. Probably, the inhibition of the CYP3A pathway in this phenotype is caused by other mechanisms, which can involve the action of proinflammatory cytokines or disturbances in serotonin turnover. Meanwhile, in rats with the high anxiety phenotype, a decrease in serotonin levels in various regions of the brain was observed in this PTSD model [19]. It is possible that elevated levels of 6βCort in stressed SM rats were associated with that previously detected decrease in the level of cerebral serotonin. Moreover, a more pronounced decrease in corticosterone levels in stressed SM rats could enhance the effects of pro-inflammatory cytokines up to neuroinflammation [20], and thus could contribute to the higher anxiety in those rats.

The feasibility of using HST for the early detection of animals susceptible and resistant to PTSD has been reported previously [21]. However, early results could not explain why HST, being the simple pharmacological test, was effective in predicting anxiety disorders.

This study has revealed the dominance of 11βHSD-2 dependent glucocorticoid metabolism in SM rats. Particularly impressive are the obtained positive correlations between the activity of 11βHSD-2 in the kidneys and indicators of anxiety behavior in the elevated plus-maze test. This opens up new ways for the translation of the results to the clinic. The impossibility of using HST in humans is obvious. However, one can assess the prospect of using 11-dehydrocorticosterone in the blood to predict the development of PTSD long before the traumatic event.

## 4. Materials and Methods

Experiments were performed on 62 Wistar male rats weighing 220–263 g. Animals were housed in standard conditions, exposed to a 12:12 h light–dark cycle, and received food and water ad libitum. All animal procedures were performed in accordance with the U.S. National Research Council Guide for the Care and Use of Laboratory Animals 2011. The experimental protocols were approved by the Ethical Committee for Animal Experiments of Institute of Immunology and Physiology, Ural Division of the Russian Academy of Sciences (# 3001-sar02-2018, Ekaterinburg, Russian Federation). The number of animals was determined based on previous studies [4,8,22]

**Figure 2:**
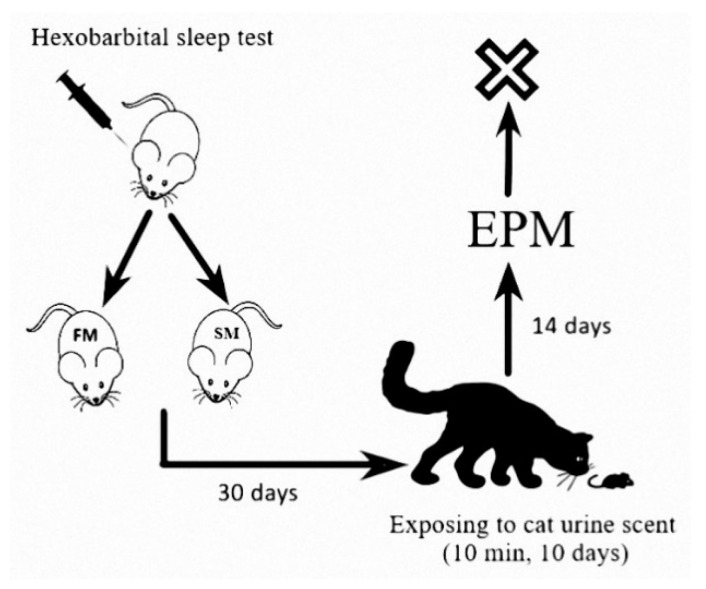
Experimental design. EPM—elevated plus maze test.

The hexobarbital solution was prepared on the day of the experiment and administered i.p. at a dose of 60 mg/kg 30 days prior to traumatic stress. The sleep time after hexobarbital (HST) administration was designated as the time between the injection of hexobarbital and recovery of the righting reflex. The righting reflex was defined as the ability of the animal, after being placed on its back on a flat surface, to turn over by 180° three times within 15 s (Figure 2).

Based on the results of the HST test, rats were divided into two groups: FM (sleep duration <15 min, *n* = 42, 68% of the study subjects) and SM (sleep duration≥ 15 min, *n* = 20, 32% of the study subjects), reflecting the intensity of microsomal oxidation [4,8]. This ratio is consistent with the ratio of PTSD-resistant and PTSD-vulnerable rats in the total population observed in our previous study, and humans exposed to a traumatic event [19,23]

The FM and SM groups were then divided into unstressed and stressed groups. Unstressed rats rested throughout this period. The stressed subgroups were exposed to cat urine scent (PSS) for 10 min daily for 10 days followed by 14 days of rest under stress-free conditions [24].

The behavior of all rats was evaluated with an elevated plus maze test, using the standard elevated plus maze (EPM) test apparatus TS0502-R3 (OpenScience, Russia). Recorded variables included the time spent in open and closed arms of the maze and the number of entries into the open and closed arms [25,26]. The video system SMART with SMART 3.0 software was used. Based on these measurements, an anxiety index [27] was calculated: AI = 1 − {[(time in open arms/time on maze) + (number of entries into open arms/number of all entries)]/2}.

Between 11.00 a.m. and 1.00 p.m. on the day following the behavioral test, rats were sacrificed by decapitation under ether anesthesia, and samples of trunk blood and adrenal glands were collected. Blood was transferred to sterile glass tubes containing K2 EDTA, rotated, and centrifuged at 40 °C and 3000 g for 10 min. Plasma was separated into aliquots, which were frozen at -80C for subsequent measurement of corticosterone.

Plasma and liver 6β-hydroxycorticosterone (6βCort), a marker of glucocorticoid metabolism, were measured using a rat 6 beta-hydroxycorticosterone (6 beta-OH-B) ELISA kit (Blue Gene Biotech, Shanghai, China) according to the manufacturer’s instruction. The assay sensitivity was 1.0 ng/mL, and the intra- and inter-assay coefficients of variation were both <5%.

Corticosterone (4-pregnen-11β,21-diol-3,20-dione) and 11-dehydrocorticosterone (21-hydroxypregn-4-ene-3,11,20-trione) (Koch-Light Laboratories Ltd., Haverhill, Suffolk, UK) were used. Standard solutions of corticosteroids (1 mg/mL) were prepared using ethyl alcohol. The working solutions of corticosteroid hormones in the concentration from 2 to 50 ng/μL were prepared from the standard solutions by a corresponding dilution with ethyl alcohol.

Determination of corticosteroid hormones in rat blood plasma was carried out using micro-column high-performance liquid chromatography (HPLC). Milikhrom-1 chromatograph (NPO Nauchpribor, Orel, Russia) was used in the work. The chromatograph was equipped with a stainless-steel chromatographic column (2 × 62 mm, Silasorb C18 SPH (5 μm)). Gradient elution was carried out with acetonitrile in water from 30 to 55% (*v*/*v*). The eluent flow rate was 100 μL/min. Detection was carried out at two wavelengths: 240 and 260 nm. These wavelengths were chosen in accordance with the absorption spectrum of steroid hormones: 240 nm is the wavelength corresponding to the maximal absorption of the majority of corticosteroid hormones, while 260 nm is the wavelength at which the absorption is about a half of the maximal level. Chromatographic information was processed with the help of CHROM software (EcoNova, Institute of Chromatography, Novosibirsk, Russia). Hormone identification was performed by comparing retention times and spectral ratios of endogenous corticosteroid hormones and synthetic preparations.

**Figure 3:**
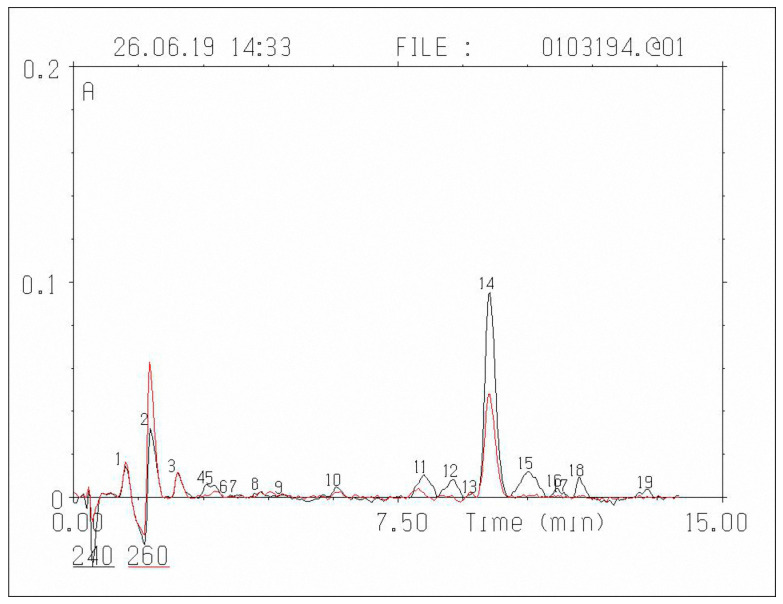
Chromatogram of rat blood plasma extract: the black line refers to absorption at 240 nm, the red line is absorption at 260 nm; N12—11-dehydrocorticosterone; N14—corticosterone.

The treatment of blood plasma. Extraction of corticosteroid hormones from blood plasma was carried out according to the procedure described previously [28], namely, 0.5 mL of blood plasma, 0.5 mL of water were poured in glass tubes 15 mL in volume. Then, 4 mL of hexane was added, and the tubes were stirred for 3 min. After that, the organic layer was removed using a water-jet pump. Then, 9 mL of chloroform was added to the aqueous phase, and extraction was carried out for 5 min. The aqueous layer was removed with a water-jet pump, and the organic layer was evaporated in portions in conical plastic tubes under the flow of nitrogen at a temperature of 40 °C. The residue was dissolved in 24 μL of a 55% solution of CH3CN in water and thoroughly mixed. The portion taken for the chromatographic analysis was 8 μL of the extract. The amount of corticosteroid hormones was determined in the units of nanograms per 1 mL of blood plasma (ng/mL). The chromatogram of rat blood plasma extract is shown in Figure 3.

Activities of 11βHSD-2 were measured according to [29]. Pieces of the renal cortex of 25–30 mg were weighed and were placed into a cooled glass homogenizer. Then, one ml of 20 mM Tris-HCl buffer, pH 8.3, containing 5 mM Mg(CH3COO)_2_, 30 mM KCl, 250 mM sucrose, and 0.5% Triton X-100 was added and homogenized thoroughly. The homogenate was centrifuged at 3000 g for 15 min at 4 °C and the supernatant was used for further analysis. A total of 100 µL of supernatant was incubated for 60 min at 37 °C in the presence of 0.2 μM corticosterone and 1.5 mM NADP in 0.1 M Na-phosphate buffer, pH 8.5. The reaction was stopped by the addition of 100 μL of acetonitrile. Then, the incubation mixture was centrifuged and 8 μL of the supernatant was analyzed by high-performance liquid chromatography (HPLC) on a “Milichrom-1” chromatograph (Nauchpribor, Russia) equipped with the analytical column (2 × 62 mm, Silasorb C18, 5 μm). Acetonitrile gradient in water (from 30 to 55% *v*/*v*) was used as the eluent. The detection wavelengths were 240 and 260 nm. Chromatographic information was processed using the CHROM program (EkoNova Institute of Chromatography, CJSC, Novosibirsk, Russia). Enzymatic activity was expressed as nanomoles of 11-dehydrocorticosterone formed per one minute in one gram of tissue (nmol min^‒1^ g^‒1^).

Statistical analysis. Data were analyzed with the SigmaPlot 12.5. Quantitative data were presented as mean ± SEM. Two-way ANOVA with Tukey post-hoc tests was used to compare all outcome measures between the groups. *p* < 0.05 was considered significant.

## 5. Conclusions

The results of the study indicate that “fast-” and “slow metabolizers” are associated with the predominating activity of various pathways of glucocorticoid metabolism. In fast metabolizers, the CYP3A-dependent pathway is activated, and in slow metabolizers, the 11βHSD-2 pathway. Our data additionally confirm the activation of the 11βHSD-2 pathway in experimental PTSD, while the resistance to PTSD can be associated with the activation of CYP3A-dependent glucocorticoid metabolism.

## Figures and Tables

**Table 1:** Laboratory indicators and behavior of fast metabolism (FM) and slow metabolism (SM) rats.

	FM (*n* = 42)		SM (*n* = 20)	
	Unstressed (*n* = 22)	Stressed (*n* = 20)	Unstressed (*n* = 8)	Stressed (*n* = 12)
Time in open arms, s	154.2 ± 24.6	258.5 ± 19.6 ***	78.5 ± 16.6 #	100.2 ± 21.4 ###
Time in closed arms, s	445.8 ± 24.6	341.5 ± 19.6 ***	521.5 ± 16.6 #	499.8 ± 21.4 ###
Entries into open arms	6.3 ± 0.6	8.7 ± 0.4 **	4.2 ± 0.5 #	5.2 ± 0.6 ###
Entries into closed arms	7.8 ± 0.8	10.6 ± 0.5 **	5.4 ± 0.6 #	10.0 ± 0.9 **
Anxiety index	0.65 ± 0.02	0.56 ± 0.02 **	0.71 ± 0.02 #	0.74 ± 0.02 ###
Grooming	2.91 ± 0.55	2.63 ± 0.47	1.0 ± 0.3	2.5 ± 0.42
Cort, nM/l	197.4 ± 18.6	135.0 ± 13.1 *	270.4 ± 37.1 #	154.3 ± 25.3 **
11Dehydro Cort, nM/l	1.3 ± 0.2	3.6 ± 0.8	5.5 ± 1.6 ###	6.5 ± 1.9
6βCort in liver, nM/g	0.28 ± 0.01	0.25 ± 0.02	0.14 ± 0.04 ##	0.24 ± 0.04 *
6βCort in blood, nM/l	0.39 ± 0.05	0.65 ± 0.1 **	0.45 ± 0.04	0.52 ± 0.03
11βHSD-2, nmol min–1 g–1	9.73 ± 0.46	10.07 ± 0.94	13.45 ± 0.67 ##	11.26 ± 0.96

Values are means ± SE. * Significantly different from unstressed rats; * *p* < 0.05; ** *p* < 0.01; *** *p* < 0.001. # Significantly different from fast metabolizers; # *p* < 0.05; ## *p* < 0.01; ### *p* < 0.001.

## Abbreviations

11Dehydro Cor	11-dehydrocorticosterone (endogenous corticosteroid)
11βHSD-2	11β-hydroxysteroid dehydrogenase type 2 (enzyme)
6βCort	6 beta-hydroxycorticosterone (endogenous corticosteroid)
AI	anxiety index
ANOVA	analysis of variance (collection of statistical models)
CYP3A	cytochrome P450 (enzyme)
EDTA EPM	ethylenediaminetetraacetic acid (chemical) elevated plus maze test
FM	fast metabolizers
HPLC	high performance liquid chromatography (method).
HST	hexobarbital sleep test
PSS	predator scent stress
PTSD	posttraumatic stress disorder
SM	slow metabolizers
